# Antibiotic therapy and the gut microbiome: Investigating the effect of delivery route on gut pathogens

**DOI:** 10.1021/acsinfecdis.1c00081

**Published:** 2021-04-12

**Authors:** Stephen A. Kelly, Jonathan Nzakizwanayo, Aoife M. Rodgers, Li Zhao, Rebecca Weiser, Ismaiel A. Tekko, Helen O. McCarthy, Rebecca J. Ingram, Brian V. Jones, Ryan F. Donnelly, Brendan F. Gilmore

**Affiliations:** 1 School of Pharmacy, Queen’s University Belfast, 97 Lisburn Road, UK, BT9 7BL; 2 Department of Biology & Biochemistry, University of Bath, Claverton Down, Bath, UK, BA2 7AX; 3 Department of Biology, Maynooth University, Maynooth, Co. Kildare, Ireland, W23 F2K8; 4 Microbiomes, Microbes and Informatics Group, Organisms and Environment Division, Cardiff School of Biosciences, Cardiff University, UK, CF10 3AX; 5 Department of Pharmaceutics and Pharmaceutical Technology, Faculty of Pharmacy, Aleppo University, Aleppo, Syria; 6 Wellcome-Wolfson Institute for Experimental Medicine, School of Medicine, Dentistry and Biomedical Sciences, Queen’s University Belfast, 97 Lisburn Road, UK, BT9 7BL

**Keywords:** Antibiotic, Dysbiosis, Gut, Microbiome, Pathogen, Route of administration

## Abstract

The contribution of the gut microbiome to human health has long been established, with normal gut microbiota conferring protection against invasive pathogens. Antibiotics can disrupt the microbial balance of the gut, resulting in disease and the development of antimicrobial resistance. The effect of antibiotic administration route on gut dysbiosis remains under-studied to date, with conflicting evidence on the differential effects of oral and parenteral delivery. We have profiled the rat gut microbiome following treatment with commonly-prescribed antibiotics (amoxicillin and levofloxacin), via either oral or intravenous administration. Fecal pellets were collected over a 13-day period and bacterial populations analysed by 16S rRNA gene sequencing. Significant dysbiosis was observed in all treatment groups, regardless of administration route. More profound dysbiotic effects were observed following amoxicillin treatment than with levofloxacin, with population richness and diversity significantly reduced, regardless of delivery route. The effect on specific taxonomic groups was assessed, revealing significant disruption following treatment with both antibiotics. Enrichment of a number of groups containing known gut pathogens was observed, in particular with amoxicillin, such as the family Enterobacteriaceae. Depletion of other commensal groups was also observed. The degree of dysbiosis was significantly reduced towards the end of the sampling period, as bacterial populations began to return to pre-treatment composition. Richness and diversity levels appeared to return to pre-treatment levels more quickly in intravenous groups, suggesting convenient parenteral delivery systems may have a role to play in reducing longer term gut dysbiosis in the treatment of infection.

The importance of the gut microbiome in contributing to human health has long been established, with perturbations linked to a number of disease states, including cardiovascular disease^1,2^, liver disease^3,4^, obesity and diabetes^5–7^, as well as altered brain function^8,9^ and mental illness^10,11^. The normal gut microbiota confers protection to the host against invasive pathogenic species, in a phenomenon referred to as ‘colonization resistance’^12^. This occurs through a number of mechanisms, including direct killing, competition for limited nutrients, and enhancement of immune responses^13^.

A healthy gut microbiome represents a highly diverse community, estimated to exceed 10^14^ microorganisms, with functions ranging from carbohydrate fermentation and vitamin synthesis, to immune system development and perhaps even nervous system functionality^14–16^. It is largely accepted that a healthy gut microbiome is both balanced and diverse, with major constituents including species from the phyla Firmicutes (such as *Lactobacillus* spp.) and Bacteroidetes (such as *Bacteroides* spp.), as well as Actinobacteria (e.g. *Bifidobacterium bifidum*) and representatives of the phyla Proteobacteria, Fusobacteria, and Verrucomicrobia^15,17,18^. The resilience of a microbiota, that is, its capacity to return to equilibrium following perturbations, also appears to be associated with higher diversity^16^.

Changes to the gut microbiota may result in both harmful and beneficial effects, and what constitutes a healthy gut microbiome is still under investigation. The effect of antibiotic therapy on the gut microbiome is a well studied phenomenon^19^. The gut has been identified as a major reservoir for antimicrobial resistance genes, which can subsequently be passed on in the feces^20^. A number of studies have highlighted the rapid enrichment of antibiotic resistance genes (ARGs) as a direct result of antibiotic use, contributing to the increasingly serious global health crisis of antimicrobial resistance (AMR) development^21–23^. In addition to AMR development, antibiotic administration can alter the microbial composition of the gut, resulting in the loss of colonization resistance and rendering the host susceptible to colonization by opportunistic pathogens^24^.

This can have specific deleterious effects on commensal microbes, as well as leading to significant proliferation of disease-causing bacteria and fungi. A longitudinal study involving human participants showed treatment with ciprofloxacin resulted in significant reductions in taxonomic richness and diversity within days of antibiotic administration^25^. Treatment with fluoroquinolones and β-lactams has also been shown to reduce microbial diversity by 25%, as well as significantly altering the Bacteroidetes:Firmicutes ratio^26^. The overall microbial load was in fact increased with antibiotic therapy, doubling in the case of β-lactam treatment, removing sensitive microorganisms and creating conditions in which resistant microbes proliferate and dominate^26^.

Similar depletion of protective commensals was observed by Palleja and co-workers, who observed that antibiotic therapy resulted in reductions of *Bifidobacterium* species and blooms of enterobacteria and other pathobionts, such as *Enterococcus faecalis* and *Fusobacterium* species^27^. This study once again highlighted the importance of a healthy gut microbiome, with the gut microbiota of healthy young adults showing greater resilience to short-term antibiotic intervention^27^. Similar pathogenic blooms were observed with vancomycin exposure in a simulated human intestinal microbial ecosystem, resulting in proliferation of opportunistic pathogens such as *Achromobacter, Klebsiella* and *Pseudomonas* species^28^.

Prior antibiotic therapy is one of the most important risk factors for colonization with *Clostridium difficile*, a spore-forming bacterium that colonizes the large intestine, causing colitis^29,30^. Clindamycin therapy has been shown to have a considerable negative impact on the intestinal microbiota, reducing resistance to colonization by pathogens and resulting in *C. difficile*-induce colitis^31,32^.

The administration route of antibiotics may also have an effect on the extent of microbial imbalance and impaired microbiota in the gut, known as dysbiosis. Work by Zhang and co-workers showed IV delivery of antibiotic significantly reduced or delayed the development of AMR, in the feces of mice inoculated with microorganisms harboring known ARGs, compared to oral therapy^33^. The differential effects of antibiotic delivery route on the gut microbiome has been similarly highlighted in other studies, with significantly different bacterial colonization and AMR profiles in orally treated animals compared with parenteral delivery^34–36^.

Whilst there are relatively few studies examining the effect of antibiotic administration route on gut dysbiosis to date, there is conflicting evidence within this limited literature^20^. A number of recent studies have shown the dysbiotic effects of parenteral antibiotic delivery on the gut microbiota^37,38^. These findings do however challenge the theory that parenterally-delivered antibiotic does not reach the gut, thereby sparing the gut microbiome, with one study in particular finding no significant differences in gastrointestinal (GI) concentration of florfenicol following either oral or intramuscular delivery^39^.

In this study, we examined the dysbiotic effect of a single dose of antibiotic on the overall gut microbiome of Sprague-Dawley rats. Commonly-prescribed antibiotics from two different classes were chosen as examples, one β-lactam (amoxicillin) and one fluoroquinolone (levofloxacin), to investigate the effect of delivery route on the gut microbiome. Each antibiotic was delivered orally and by intravenous (IV) injection, to directly compare the effect of delivery route on gut microbial populations. As well as considering the effect of antibiotic administration on gut microbial community structures and diversity, statistical analysis is used to assess the effects of antibiotic on specific taxonomic groups, including those with high numbers of gut pathogens. These changes are explored to compare the effects of enteral and parenteral antibiotic therapy, and determine the importance of delivery route in avoiding dysbiosis in the gut microbiome.

## Results and Discussion

### Sampling and sequencing summary

Individually caged Sprague-Dawley rats were either treated with a single dose of amoxicillin sodium (orally or IV), levofloxacin hydrochloride (orally or IV), or left untreated. Fecal pellets were collected at various timepoints over a 13-day period, including several days before treatment and for eight days post-treatment. Samples were collected on Days 1 and 2, before treatment on Day 5 (5a), 8 h post-treatment on Day 5 (5b), as well as on Days 6, 7, 8, 9, 12 and 13. Each of the six treatment group consisted of six rats, resulting in the collection of 360 individual samples in total. DNA was extracted and prepared from each sample as described, and amplicons of the V4 region of the 16S rRNA gene was sequenced to allow for downstream taxonomic analysis. DNA was extracted from all 360 collected samples, with 356 resulting in successful sequencing, (n=6 for all groups except Oral treatment on Day 7 and IV treatment on Day 9 for both antibiotics, where n=5). The sequencing was done in two batches, the amoxicillin and the levofloxacin groups. Samples were pooled per group to allow multiplexing in a single sequencing run. Total reads prior to quality checking were 9,616, 786 in the amoxicillin group, and 9, 672, 861 in levofloxacin. These were reduced to 3, 281, 118, and 2, 841,709 high quality sequences, respectively; made up of 200, 721 and 202, 947 unique sequences, respectively. The sequences were subsequently binned into OTUs at > 97% identity level.

### Gut microbiome in untreated samples

Prior to antibiotic treatment, similar taxonomic profiles were observed for the gut microbiomes of all animals at the phylum level. As expected, members of the phylum Firmicutes predominated, constituting mean proportions of 77.7 to 82.3% for each group. This was followed by Bacteroidetes (12.0 to 15.3%), Actinobacteria (1.5 to 2.0%) and Proteobacteria (1.1 to 1.9%), with the remainder made up of Cyanobacteria, Verrucomicrobia, and Deferribacterota. Unclassified sequences constituted a low proportion of overall reads at this taxonomic level, ranging from 0.3 to 3.6% across all groups. This proportion increased following antibiotic therapy in some samples, increasing to a maximum of 4.6% in levofloxacin samples, and 14.6% with amoxicillin, where greater dysbiosis was observed. The observed dominance of the Firmicutes and Bacteroidetes phyla in untreated samples, is in keeping with previous studies which have reported approximately 90% relative abundance of both phyla in the gut microbiota of the wild-type murine gut^40,41^.

### Overall population shifts - Phylum level analysis

**Figure 1:**
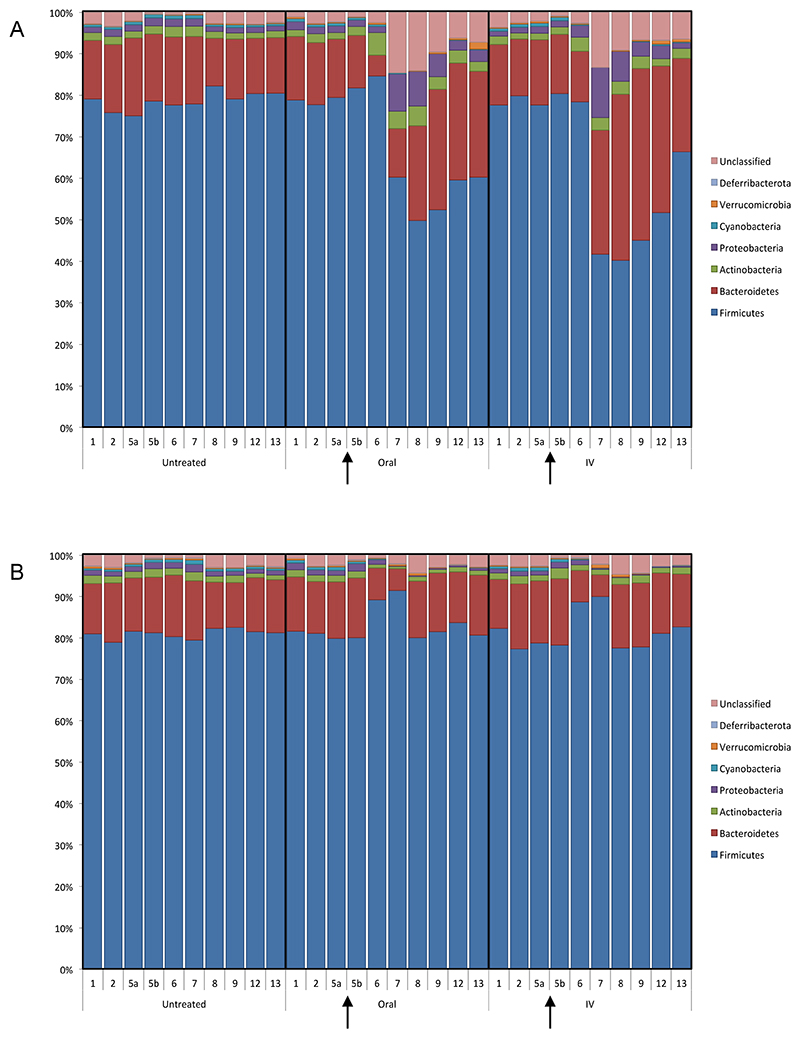
Taxonomic analysis showing population shifts in bacterial phyla over a 13-day time period before and after A. Amoxicillin, and B. Levofloxacin administration. Treatment times (Day 5), are denoted by an arrow for each treated group.

As can be seen from Figure 1, shifts in bacterial population were observed following antibiotic administration in all treatment groups, regardless of antibiotic or administration route. Initial dysbiosis is observed 24 hours post treatment in all treatment groups, with populations remaining relatively consistent throughout in untreated samples. Population shifts observed following IV administration of both antibiotics suggests antibiotic reaches the gut, even following parenteral delivery, and does so in sufficient quantities to bring about substantial dysbiosis in this niche.

Maximal dysbiosis is observed at around 48-72 h post treatment. As such, statistical differences before and after treatment were determined using groups from Day 5a and Day 7. Following treatment with amoxicillin, reductions were observed in the most dominant Phylum, Firmicutes, following both oral and IV administration (Figure 1A). Interestingly, in the second most dominant Phylum, Bacteroidetes, changes were quite different between the oral and IV groups. Following oral treatment, there was no significant difference between Day 5a and Day 7, whereas the proportion of Bacteroidetes was significantly increased following IV administration (p < 0.0001), increasing from 15.7 to 29.8%. All other population shifts following treatment with amoxicillin were consistent between oral and IV administration groups, with increases in Actinobacteria and Proteobacteria, and decreases in Cyanobacteria and Verrucomicrobia. The proportion of Proteobacteria increased markedly following amoxicillin administration, experiencing almost a 6-fold increase following oral administration (1.6 to 9.0%), and a 7.5-fold increase after IV antibiotic (1.6 to 12.0%). This may be of particular relevance to bacterial infections of the gut, with members of this phylum in particular known to be causative organisms in a number of gastrointestinal infections, however this is difficult to confirm, and more robust classification at lower taxonomic levels would be required. Proteobacteria are known to increase in abundance after antibiotic treatment as they often exhibit more antimicrobial resistance than other groups, possibly explaining the increased abundance observed following amoxicillin administration.

Bacterial shifts are also observed following treatment with levofloxacin, albeit to a lesser degree (Figure 1B). It is worth noting that the profile of population shift, as well as being lower in severity, is also quite different at phylum level in comparison to amoxicillin. Unlike with amoxicillin, the proportion of Firmicutes increased following levofloxacin therapy.

While members of Phylum Firmicutes increasingly dominated the gut microbiome, accounting for around 90% of all bacteria present, members of Bacteroidetes, Proteobacteria, Cyanobacteria, and Deferribacterota all decreased following levofloxacin treatment. These data correlate closely with a recent study which compared the differential effects of levofloxacin and a number of β-lactam antibiotics (meropenem, cefoperazone/sulbactam, and aztreonam) on the gut microbiota^42^, which found an increase in Firmicutes and a decrease in Bacteroidetes following levofloxacin treatment. Similarly, work by Ziegler and co-workers^43^ investigated the impact of levofloxacin on the gut microbiome in comparison to the β-lactams cefepime, piperacillin-tazobactam, and meropenem. In both studies, despite the antibiotics chosen being different, disruptive effects of β-lactams were much greater than levofloxacin, a finding shared by this study.

### Effect of antibiotic on OTU richness and diversity

Population richness and diversity was investigated for all treatment groups at Day 5a and following treatment at Day 7, with untreated samples at the same timepoints included as additional controls. Alpha diversity measurements included good coverage scores, the observed species richness (SOBS) and the Shannon Index for diversity, as well as rarefaction analysis as a further measure of population richness.

**Figure 2:**
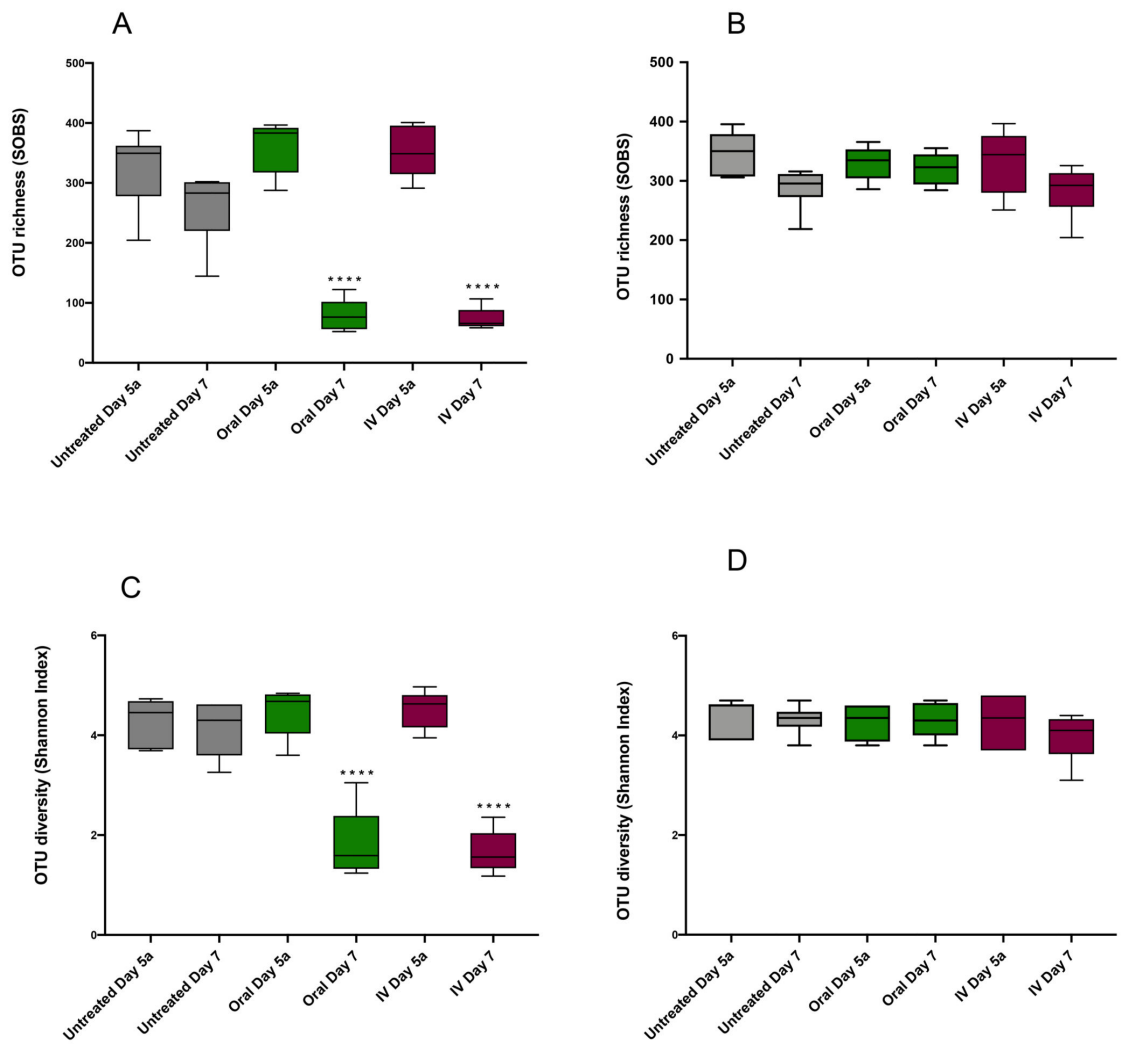
Richness and diversity analysis before (Day 5a) and after (Day 7) antibiotic administration measured by SOBS and Shannon Index A. Amoxicillin SOBS. B. Levofloxacin SOBS. C. Amoxicillin Shannon Index. D. Levofloxacin Shannon Index. **** = p<0.0001, *** = p<0.0002, ** = p<0.0021, * = p<0.0332. Good coverage score was ≥92.00% at the rarefaction, average 93.77 ± 0.99%. Significant differences in diversity indices between samples were determined by ANOVA using GraphPad Prism 8.

**Figure 3:**
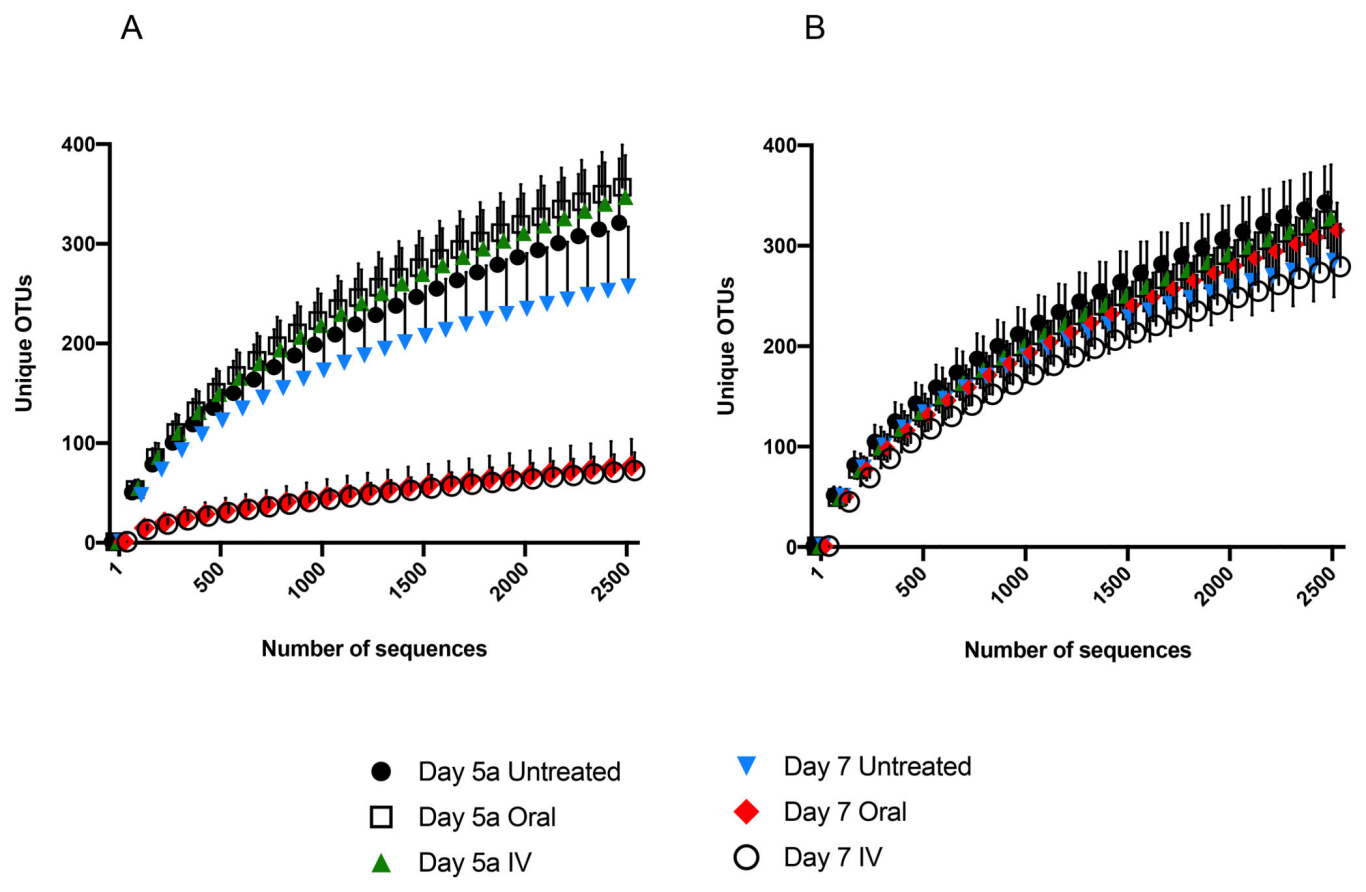
Rarefaction analysis showing the cumulative number of unique OTUs uncovered as sequencing progressed before (Day 5a) and after (Day 7) A. Amoxicillin, and B. Levofloxacin administration.

Good coverage score was ≥92.00%. Richness, the number of species in a community, was significantly decreased following both oral and IV amoxicillin (p<0.0001), with SOBS values decreasing from 361.46 ± 43.96 (Day 5a) to 78.44 ± 27.24 (Day 7) for oral dosing, and from 351.10 ± 42.02 to 73.41 ± 18.26 at the same timepoints for IV. There was no significant difference between untreated groups at these timepoints (Figure 2A). Rarefaction analysis, another measure of population richness, revealed similar trends (Figure 3A). Following the sequencing of 2500 reads, the mean number of unique Operational Taxonomic Units (OTUs) for untreated and pre-treatment samples ranged from 257.05 to 356.64. However, in oral and IV treatment groups at Day 7, the mean number of unique OTUs at 2500 sequences was just 77.04 and 72.40 respectively. In other words, as sequencing progressed, the rate of unique OTUs uncovered was significantly reduced following treatment with amoxicillin compared to untreated samples, indicating significantly lower population richness.

**Figure 4:**
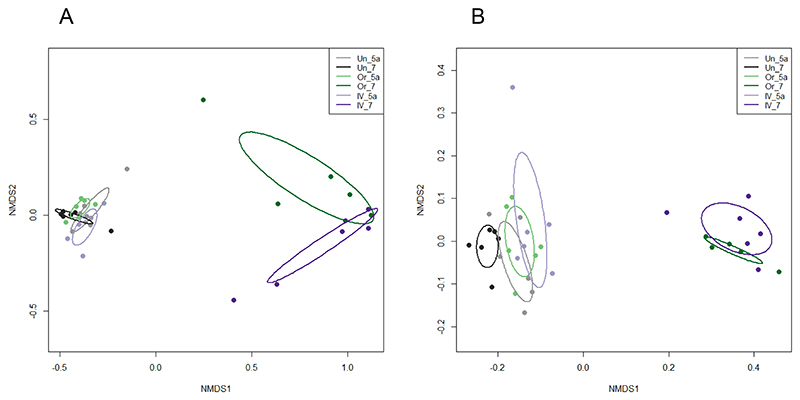
NMDS ordination of Bray-Curtis dissimilarity values for samples before (Day 5a) and after (Day 7) amoxicillin (A) and levofloxacin (B) administration. Untreated samples are also included as controls. Samples are grouped by treatment and colour coded as shown in the key: Untreated group Day 5a (Un_5a) and Day 7 (Un_7); Oral administration group Day 5a (Or_5a) and Day 7 (Or_7); IV administration group Day 5a (IV_5a) and Day 7 (IV_7). Points represent individual samples and ellipses are standard deviations of point scores for each grouping.

Shannon diversity, which also takes into account abundance and community distribution, was also significantly reduced following amoxicillin administration (p<0.0001) (Figure 2C). Shannon Index values decreased from 4.47 ± 0.49 on Day 5a to 1.80 ± 0.72 on Day 7 for oral administration (a decrease in the mean value of 2.67), and from 4.53 ± 0.57 to 1.66 ± 0.43 at the same timepoints for IV, (a decrease in the mean of 2.87). As with SOBS analysis for richness, an identical level of significance was observed in the reduction of diversity between oral and IV treatment groups, with no significant differences between untreated samples at Day 5a and Day 7. Differences in community composition between samples (Beta diversity) taken at Day 5a and Day 7 were evaluated using NMDS ordination of Bray-Curtis dissimilarity values (Figure 4). Untreated controls at Day 5a and Day 7 clustered together on the NMDS plots and were not significantly different. Samples from the antibiotic treatment groups before (Day 5a) and after (Day 7) oral and IV administration of amoxicillin (Figure 4A) and levofloxacin (Figure 4B), clustered separately on the NMDS plots and were significantly different, indicating changes in the microbiome following antibiotic administration (Supplementary Tables 1 and 2).

Two important conclusions can be drawn from these data. Firstly, antibiotic therapy, even after a single dose, significantly reduces the richness and diversity of the rat gut microbiota. Secondly, the degree of significance was the same following either oral or IV administration, suggesting that regardless of route of administration, amoxicillin has the potential to find its way into the gut, resulting in perturbations to the microbiome.

Whilst marked effects on population richness and diversity were observed following amoxicillin treatment, similar population disruption was not seen with levofloxacin. Within the levofloxacin treatment groups, similar profiles were again observed between oral and IV administration, with no significant differences in either richness or diversity as measured by SOBS and Shannon Index respectively for either group (Figure 2B and D). Rarefaction analysis also shows that richness is largely unaffected relative to amoxicillin, with all treated and untreated groups following similar trends (Figure 3B).

### Effect antibiotic treatment on specific bacterial families

**Figure 5:**
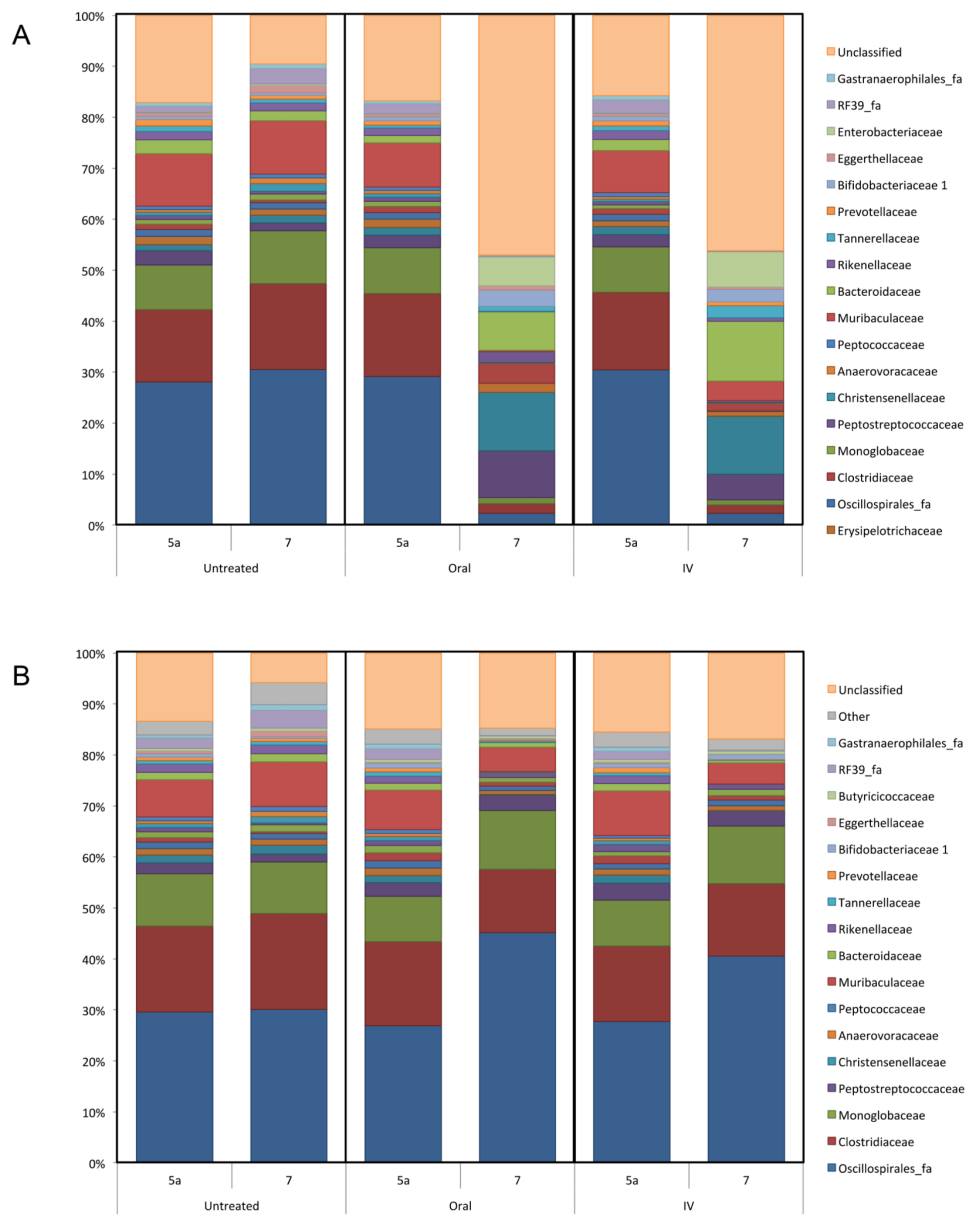
Taxonomic analysis showing initial population shifts at Family level before (Day 5a) and after (Day 7) A. Amoxicillin, and B. Levofloxacin administration.

The effect individual bacterial families was also investigated, in order to determine which groups were significantly enriched or depleted by antibiotic therapy (Figure 5). As with previous measures of dysbiosis, much greater disruption was observed with amoxicillin compared to levofloxacin. Following both oral and IV administration, amoxicillin treatment resulted in significant decreases in the proportion of Lachnospiraceae, Ruminococcaceae and Oscillospiraceae from Phylum Firmicutes, as well as Muribaculaceae from Phylum Bacteroidetes (Figure 5A). The degree of significance was the same for all of these groups for both oral and IV delivery (p<0.0001), with the exception of Muribaculaceae, in which the effect was more pronounced following oral therapy (p<0.0021 for oral versus no significant change for IV) (Table 1). Investigated dysbiotic changes down to family level allows us to predict the potential deleterious effects of antibiotic administration on the gut microbiome, even following a single dose. Members of the Lachnospiraceae family, such as *Roseburia intestinalis*, have been shown to engender protective effects via inhibition of entero-pathogens, as well as other beneficial effects in metabolic disease and inflammatory bowel disease (IBD)^44^. Other groups, depleted as a result of amoxicillin administration in this study, are known to provide protective commensal effects, guarding against the proliferation of opportunistic pathogens. Members of the Oscillospriraceae family may contribute to the formation of secondary bile acids, protecting the host against *C. difficile* infection^45^. Families of Phylum Firmicutes, whose proportion was significantly depleted by amoxicillin, have also been shown to garner beneficial effects on gut health. Members of Family Ruminococcaceae carry out important gut functions, such as resistant starch degradation^46^, while members of Oscillospiraceae have also been found to be correlated with leanness and health in humans^45^. As shown in Table 1, a number of groups were significantly enriched as a result of a single dose of amoxicillin. Perhaps the most alarming microbial shift is the enrichment of Enterobacteriaceae. The detrimental effects of Phylum Proteobacteria in the gut, of which the Enterobacteriaceae are members, has been highlighted above. A high number of Enterobacteriaceae are known gut pathogens, including *E. coli*^47^, *Klebsiella pneumoniae*^48^, *Salmonella* spp.^49^, and *Shigella* spp.^50^, resulting in considerable infection, morbidity, and indeed mortality, globally. Enterobacteriaceae are known to proliferate in the context of intestinal inflammation, overgrowing in celiac disease and colon cancer^13^. Enterobacteriaceae are enriched in IBD, such as Crohn’s Disease and Ulcerative Colitis, and may even contribute to the rise of colitis in the first instance^51,52^.

A number of groups whose proportions increased following amoxicillin administration are known to have beneficial effects. Lactobacillaceae, which was significantly enriched following treatment, improves intestinal inflammation by increasing gastrointestinal barrier, reducing the proliferation of gut pathogens in liver diseases and IBD^53^. Interestingly, this group was only enriched following oral delivery of amoxicillin, with the same effect not observed following IV administration.

A number of differences were observed between amoxicillin and levofloxacin treatment groups. As highlighted, the degree of disruption was much lower following levofloxacin administration versus amoxicillin, with Lactobacillaceae, Clostridales_vadinBB60_group_fa, and Enterobacteriaceae exhibiting no significant changes, as had been the case with amoxicillin (Figure 5B and Table 1). A number of families within Phylum Firmicutes, namely Lachnospiraceae and Oscillospiraceae, were enriched following levofloxacin treatment, an opposite effect to that seen with amoxicillin. A number of recent studies have examined gut modulation following levofloxacin therapy, with differing correlations observed, with Lachnospiraceae found to be both enriched^42,43^ and depleted^54^, following levofloxacin administration.

The increase in Lachnospiraceae abundance in this study is commensurate with findings at phylum level, in which an overall increase in the proportion of Phylum Firmicutes was observed following both oral and IV levofloxacin administration.

Overall, these examples serve to highlight the damaging effects of antibiotic administration on the gut microbiome, even following a single dose. As well as reducing the proportions of normal commensals known to exert protective and beneficial effects in the gut, antibiotic administration creates an environment in which potentially harmful groups, including known gut pathogens, can thrive. With a few minor exceptions, this appears to be the case in many instances regardless of route of administration, suggesting once again the theory that parenterally administered antibiotic avoids the gut may not be true.

The proportion of unclassified reads also increased following treatment, regardless of antibiotic or administration route. This change was more pronounced with amoxicillin than levofloxacin, with the mean percentage of unclassified reads increasing from 16.8 and 15.8% on Day 5a, to 47.0 and 46.2% on Day 7, for oral and IV administration respectively. Whilst an increase in the percentage of unclassified reads was also observed following levofloxacin administration, this change was smaller, with 14.9 and 15.5% unclassified at the Family level on Day 5a for oral and IV groups respectively, with unclassified reads constituting 20.2 and 21.1% of all OTUs on Day 8 for the same groups.

### Duration of post-treatment dysbiosis

**Figure 6:**
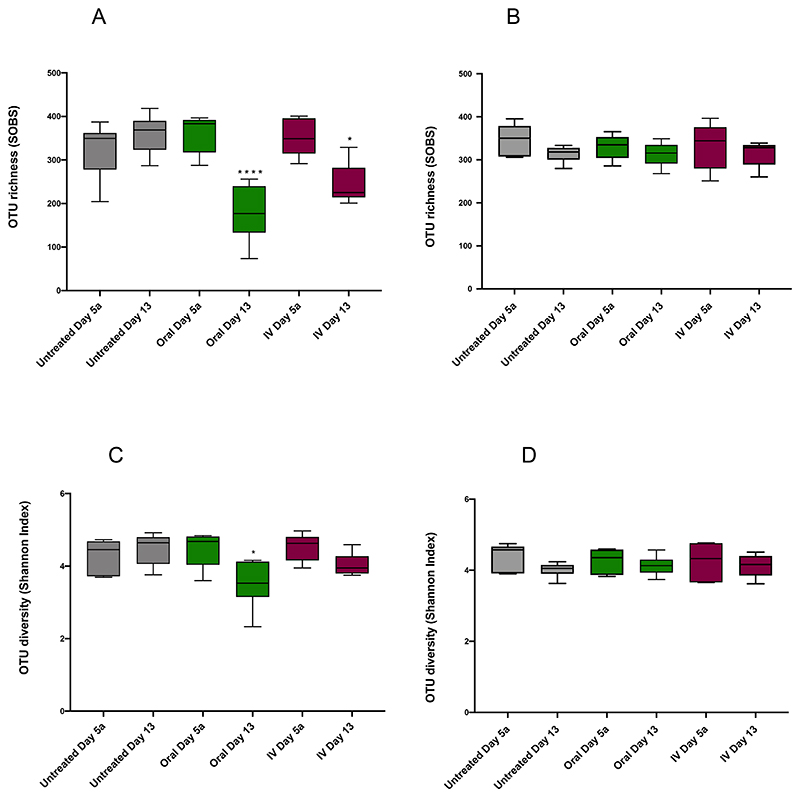
Richness and diversity analysis before (Day 5a) and 8 days after (Day 13) antibiotic administration measured by SOBS and Shannon Index A. Amoxicillin SOBS. B. Levofloxacin SOBS. C. Amoxicillin Shannon Index. D. Levofloxacin Shannon Index. **** = p<0.0001, *** = p<0.0002, ** = p<0.0021, * = p<0.0332. Good coverage score was ≥92.00% at the rarefaction, average 94.22 ± 0.72%. Significant differences in diversity indices between samples were determined by ANOVA using GraphPad Prism 8.

**Figure 7:**
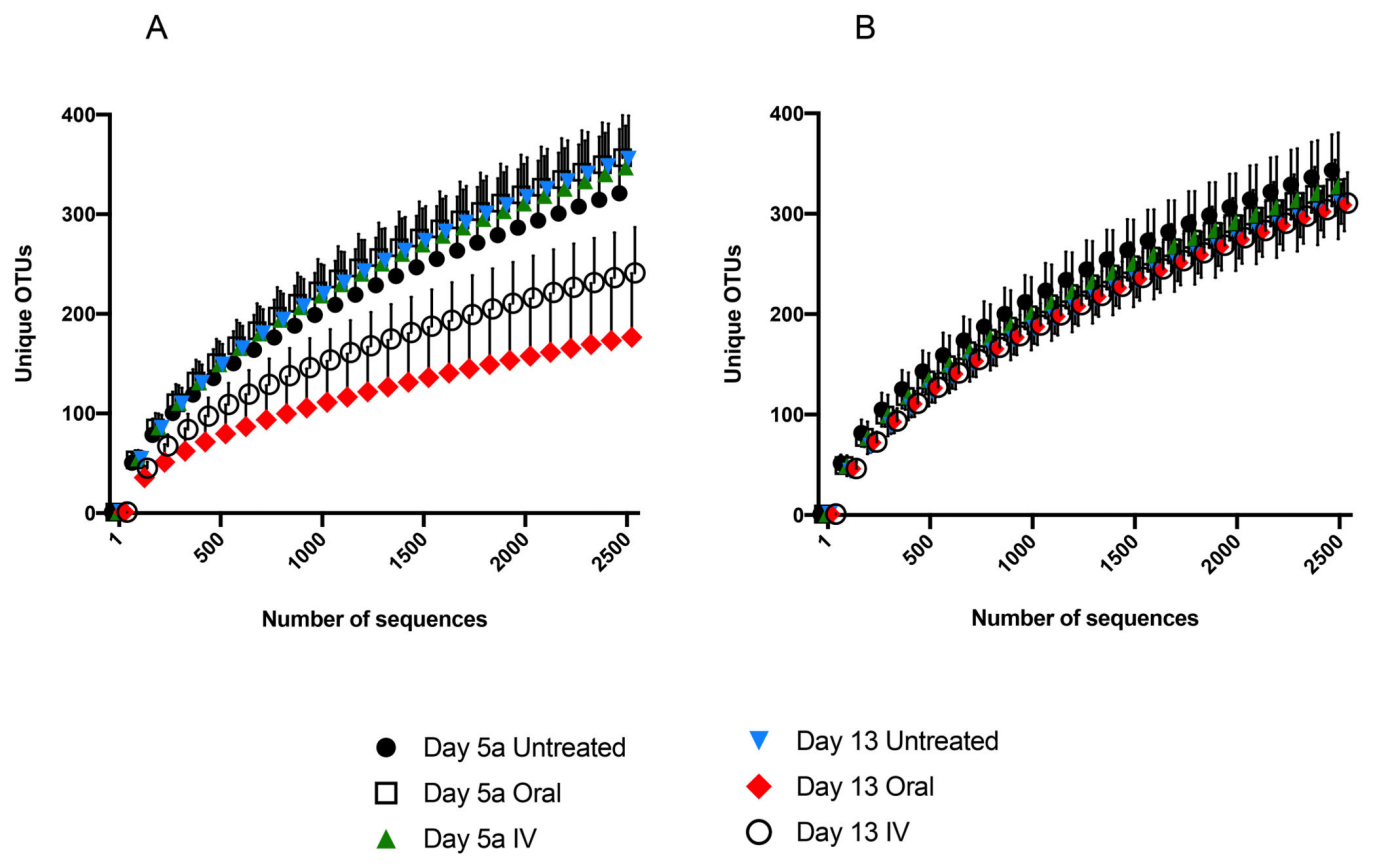
Rarefaction analysis showing the cumulative number of unique OTUs uncovered as sequencing progressed before (Day 5a) and 8 days after (Day 13) A. Amoxicillin, and B. Levofloxacin administration.

Measurement of initial dysbiosis is of high importance as it shows the acute effect of antibiotic administration, as well as highlighting potentially damaging population shifts in the gut microbiota. It is also important however to measure longer term effects of antibiotic therapy on the gut microbiome, and assess whether taxonomic breakdown returns to similar compositions observed before treatments. Fecal samples were collecting up until eight days after treatment (Day 13), and subjected to the same analysis as initial dysbiosis samples at Day 7. SOBS analysis revealed significant differences remained in animals treated with oral amoxicillin, between Day 5a and Day 13, (p<0.0001), with richness still significantly reduced (178.52 ± 64.71 at Day 13 versus 361.46 ± 43.96 at Day 5a). Richness was also significantly lower in the IV amoxicillin group at Day 13 (244.27 ± 46.75 versus 351.10 ± 42.02 at Day 5a), albeit to a lesser degree (p<0.0332) (Figure 6A). Similar trends in richness were observed with rarefaction analysis (Figure 7A). Rarefaction analysis revealed a greater number of unique OTUs uncovered as sequencing progressed at Day 13, in comparison to Day 7, for animals in both the amoxicillin oral and IV treatment groups. As with SOBS analysis, greater richness was observed in IV-treated animals, as evidenced by the greater number of unique OTUs uncovered as sequencing progressed.

OTU diversity, as measured by the Shannon Index, appears to have recovered more quickly than richness measures following the same amoxicillin treatments. However, diversity was still significantly reduced at Day 13 (3.51 ± 0.66 versus 4.47 ± 0.49 at Day 5a), albeit to a lesser degree (p<0.0032). There was no significant difference in diversity following IV delivery of amoxicillin between Day 5a and Day 13 (Figure 6C).

A number of interesting conclusions can be derived from these data. Firstly, the gut microbiome is still significantly disrupted eight days after treatment with a single dose. Secondly, dysbiosis persists following both oral and IV administration, although the gut microbiome of animals in the IV treatment group appears to be approaching levels of richness and diversity seen in pre-treated and untreated groups more quickly than in oral treatment groups. The apparent faster return to pre-treatment levels of richness and diversity may highlight a potential advantage for parenterally delivered antibiotics, in that their dysbiotic effects may not be as long-lived as with oral treatment. IV antibiotic administration is not as convenient as oral therapy however, necessitating specially-trained personnel, and giving rise to issues such as needle-phobia and introduction of infection. More user friendly parenteral delivery systems may have a role to play in this area, such as microneedle arrays, which have already demonstrated potential for this purpose^55,56^.

There were no significant differences in OTU richness following levofloxacin treatment between Days 5a and 13, for either oral or IV administration (Figure 6B). These results were corroborated by rarefaction analysis, with similar profiles observed for all groups (Figure 7B). Similarly, there were no significant differences in OTU diversity in any levofloxacin treatment groups between Days 5a and 13, as measured by the Shannon Index (Figure 6D). These results again highlight the lower overall dysbiotic effects observed with levofloxacin in this study, compared to amoxicillin.

Richness and diversity results are mirrored somewhat by analysis of changes in proportions of bacterial families after antibiotic administration (Table 1). Following oral amoxicillin treatment, Lachnospiraceae and Ruminococcaceae were significantly depleted at Day 13, while only Ruminococcaceae were still significantly affected at Day 13 following IV amoxicillin administration (p<0.0001). It is worth noting that Lachnospiraceae was significantly increased in the untreated group of the amoxicillin study at Day 13 compared to Day 5a, the only such example of a significant change in a specific family in untreated animals. No significant differences were observed at Day 13 in the other families which were significantly affected at Day 7, namely Oscillospiraceae, Lactobacillaceae, Clostridales_vadinnBB60_group_fa, Muribaculaceae, and Enterobacteriaceae. These data highlight that whilst gut dysbiosis is still observed eight days after a single dose of amoxicillin, the level of disruption is greatly reduced in comparison to the peak dysbiosis observed after treatment.

There were no significant differences in the proportions of bacterial families in either levofloxacin treatment group at Day 13, with any groups significantly changed at Day 7 appearing to return to pre-treatment levels.

**Figure 8:**
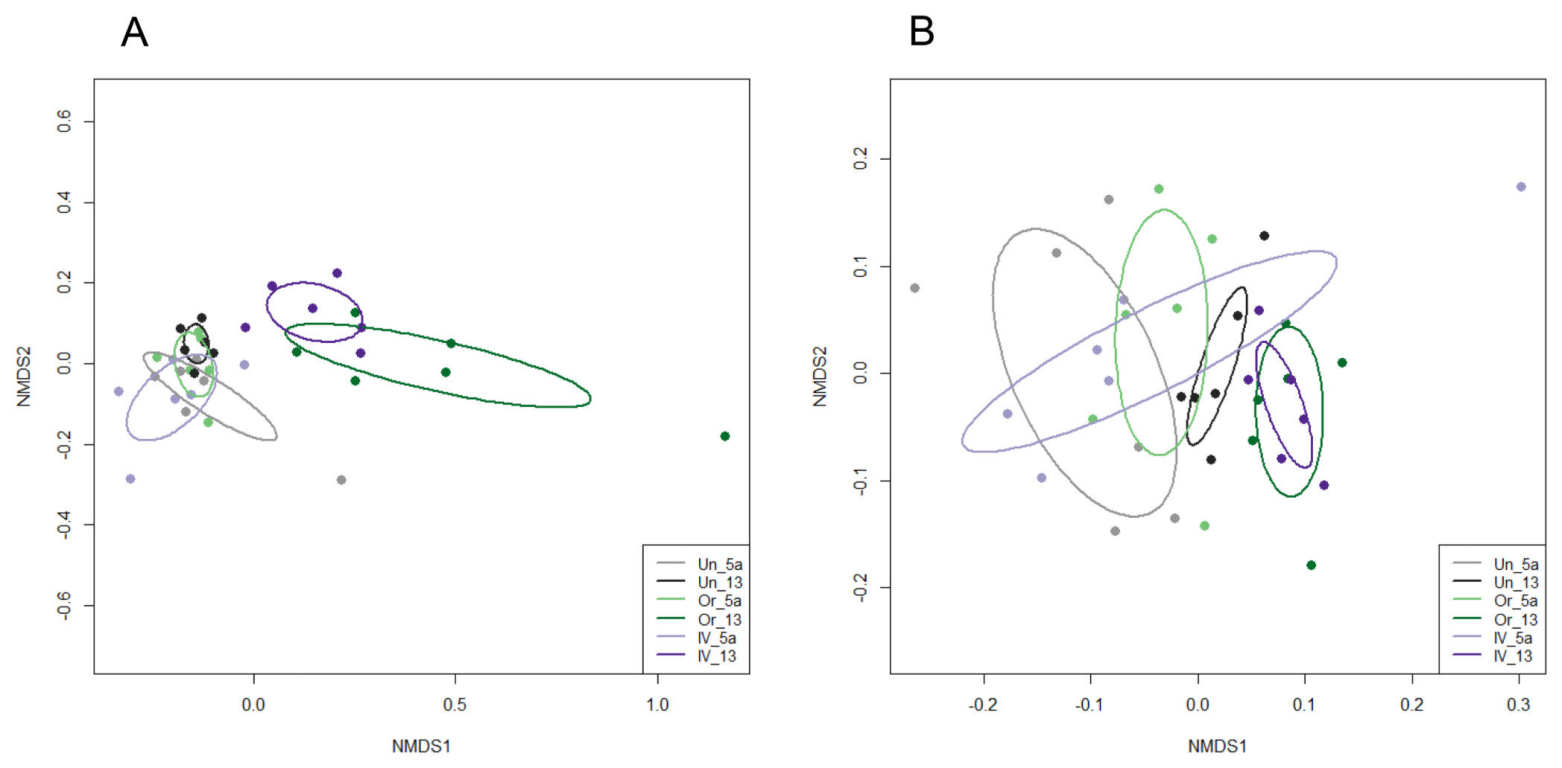
NMDS ordination of Bray-Curtis dissimilarity values for samples before (Day 5a) and after (Day 13) amoxicillin (A) and levofloxacin (B) administration. Untreated samples are also included as controls. Samples are grouped by treatment and colour coded as shown in the key: Untreated group Day 5a (Un_5a) and Day 13 (Un_13); Oral administration group Day 5a (Or_5a) and Day 13 (Or_13); IV administration group Day 5a (IV_5a) and Day 13 (IV_13). Points represent individual samples and ellipses are standard deviations of point scores for each grouping.

Beta diversity analysis of sample groups at Day 5a and Day 13 revealed that for the amoxicillin treatment group, oral and IV treatment samples were still significantly different from Day 5a samples and clustered separately on an NMDS plot (Figure 8A; Supplementary Table 3), although there were reduced distances between the clusters when compared to Day 7 (Figure 4A). It should also be noted that although untreated controls clustered together, Day 5a and Day 13 groupings were deemed significantly different, revealing natural variation in the microbiome over this time period (Figure 8A; Supplementary Table 3). For the levofloxacin treatment group, there were no significant differences between Day 5a and Day 13 for untreated, oral or IV treatment groups, suggesting that microbiome composition was returning to baseline after antibiotic administration (Figure 8B; Supplementary Table 4).

This study highlights the significant dysbiotic effects that occur following a single dose of antibiotic alone. Notably, this dysbiosis appears to persist to some extent with amoxicillin, up to at least eight days after treatment, when fecal sampling ceased. Work by Jakobsson and co-workers have shown that microbiota can remain perturbed for an extended period of time following multiple antibiotic doses, with gut dysbiosis and antimicrobial resistance determinants present in high levels up to four years after a one-week treatment course^57^. It appears there is scope to expand this study in a number of ways, in order to further assess the differences in dysbiosis caused between oral and IV delivery. The number of doses could be increased to determine if dysbiosis becomes even more pronounced initially, as well as extending sample collection for a longer period post-treatment, in order to more fully appreciate the longer term effects of antibiotic administration by different delivery routes.

## Conclusion

In this study we examined the effect of antibiotic administration route on the level of dysbiosis in the murine gut, focussing on two commonly prescribed antibiotics, amoxicillin and levofloxacin. Significant microbial perturbations were observed following a single dose of either antibiotic, although effects were more pronounced with amoxicillin. Interestingly, there was little difference in initial dysbiosis between oral and IV delivery, disproving the hypothesis that parenterally delivered antibiotic does not reach the gut to the same extent as with oral delivery, thereby causing lower levels of disruption. In particular with amoxicillin, both oral and IV delivery resulted in significantly decreased OTU richness and diversity. Amoxicillin delivery by both routes of administration also resulted in the depletion of perennially protective groups of bacteria, as well as the enrichment of Enterobacteriaceae, a family containing a multitude of species known for their propensity to act as gut pathogens and causative agents in a number of gastrointestinal infections. Richness and diversity levels appear to return to pre-treatment levels more quickly following IV administration, suggesting convenient parenteral delivery systems may have a role to play in reducing gut dysbiosis in the treatment of infection. Overall, this study represents an important addition to otherwise scant literature knowledge of the effects of different antibiotic administration routes on the gut microbiome, highlighting the potential role delivery route may have on reduction of long term dysbiosis and resulting AMR development.

## Methods

### Antibiotic administration and sample collection

All *in vivo* experiments undertaken involved 10-week-old female Sprague-Dawley rats, caged individually. Treatment groups were as follows: untreated (an untreated group was included for each antibiotic experiment), 50 mg/kg amoxicillin via oral gavage, 50 mg/kg amoxicillin via IV injection, 46 mg/kg levofloxacin hydrochloride via oral gavage, and 46 mg/kg levofloxacin hydrochloride via IV injection (n=6 for each group). Each treatment involved a single dose of antibiotic on day 5. Fecal pellets were collected from the cages of each animal on days 1, 2, 5a (before treatment), 5b (8 h post-treatment), 6, 7, 8, 9, 12, and 13, transferred to sterile vials and frozen immediately at −80 °C. All stool samples were removed from the cage at each timepoint to ensure subsequent stools were collected on the day they were produced.

### Metagenomic DNA extraction

Frozen fecal pellets were thawed on ice and ~200 mg of each sample was processed for extraction of total metagenomic DNA using the DNeasy PowerSoil Kit (QIAGEN, Germany) according to the manufacturers instructions. For each batch of DNA extraction, a negative control or ‘kitome’, was included in order to check any potential contamination introduced by the DNA extraction kit and laboratory reagents and environment. A cocktail of well defined Gut Microbiome Whole cell Mix (ATCC® MSA-2006™) was also processed and used as positive control.

### 16S rRNA gene sequencing

DNA extracts were quantified using Qubit® dsDNA assays (Thermo Fisher Scientific), and DNA purity established on the basis of A260 measurements from a Nanodrop ND-2000 spectrophotometer. The DNA was used as template for PCR using the 515F–806R (Golay-barcoded) primer pairs targeting the V4 region of the 16S SSU rRNA according to protocols presented by Caporaso and co-workers^58,59^. The forward primer was common to all samples whereas barcoded reverse primer 806R differed for each individual sample. The PCR was performed using the *KAPA HiFi HotStart* DNA Polymerase (KAPABIOSYSTEMS, London, UK). After amplification, the PCR products were detected, purified, quantified and pooled together in equimolar amounts to facilitate multiplexing. No background amplification of DNA was observed in kitome controls. The pooled library was adjusted to 10 nM final concentration, then submitted for sequencing alongside 100uM stocks of sequencing primers according to Caporaso and co-workers^58^. The sequencing was performed on the Illumina Miseq sequencing platform using paired end reads and the MiSeq 2 × 300 cycles v3 kit. All sequencing was performed at the Teagasc sequencing facility (Teagasc Food Research Centre, Moorepark, Co. Cork, Ireland) in accordance with standard Illumina protocols.

### Bioinformatics analysis

The sequencing data were analysed using the open source software Mothur v.1.44.3^60^, according to the Mothur MiSeq SOP^61^. Briefly, the paired sequence reads were assembled, demultiplexed into sample groups, then filtered to the expected V4 region length by trimming primer and low-quality sequences. The sequences were aligned to the 16S rRNA V4 database, which was extracted from the SILVA 16S rRNA reference file release 138^62,63^. Possible chimeric sequences were removed by the UCHIME algorithm^64^, and their taxonomic classification was established using the SILVA SSU NR v138 database. The sequences were further clustered into OTUs with a cutoff of 97% 16S rRNA gene similarity (0.03 distance). To control for the differences in sequencing depth, each sample was rarefied to the minimum sequence number of the smallest library size across the samples (n=2562).

Subsequently, Alpha diversity metrics including Good’s coverage, Shannon diversity estimator, SOBS richness estimator, and rarefaction curves, were calculated using Mothur. To control for the sampling effort, each sample was rarefied to the minimum sequence number of the smallest library size across the samples. Significant differences in diversity indices between samples were determined by ANOVA using GraphPad Prism 8.

Beta diversity analysis was performed using R statistical software^65^. To visualise differences in bacterial community composition between treatment groups, non-metric multidimensional scaling (NMDS) based on Bray-Curtis dissimilarity values was used (vegan package). Permutational ANOVA (PERMANOVA, adonis function in vegan package) and post-hoc pairwise PERMANOVAs with corrections for multiple testing (RVAideMemoire package, “BY” adjustment) were performed to determine statistical differences between groups. The betadisper function (vegan package) was used to test homogeneity of group dispersions (variances) as a condition of PERMANOVA. Statistical significance was determined at p<0.05. To visualise microbiota profiles, stacked bar charts of OTU relative abundances within each sample were generated using MS Excel. Command scripts and oligo files showing individual samples barcodes used in this study are provided as Supplementary information 1, in addition to R scripts used for Beta-diversity analysis. Metagenome sequence data from this study were submitted to the NCBI Sequence Read Archive (SRA) under the accession number: PRJNA702193.

### Statistical analysis

Statistical analysis was carried out using GraphPad Prism 8 software. Statistical differences between groups were determined by using ANOVA tests to compare differences from a number of groups, followed by Tukey’s post tests to compare the mean of each group to all other groups. For comparison of bacterial families, Shapiro-Wilk and Kolmogorv-Smirnov normality tests were used to determine normal distribution of datasets. Statistical differences for groups whose data were not normally distributed were determined using the non-parametric Kruskall-Wallis test, with Dunn’s post tests to compare the mean of each group to all other groups.

## Supplementary Material

Supplementary information

## Figures and Tables

**Table 1:** Changes in proportions of bacterial families following antibiotic administration, showing either increase or decrease, along with significance values, for each change. Each timepoint is shown in comparison to the corresponding samples before treatment (Day 5a). Statistical differences between groups were determined by using ANOVA tests to compare differences from a number of groups, followed by Tukey’s post tests to compare the mean of each group to all other groups for normally distributed data sets, and Kruskal-Wallis, followed by Dunn’s post tests (**** = p<0.0001, *** = p<0.0002, ** = p<0.0021, * = p<0.0332).

Phylum	Family	Amoxicillin					Levofloxacin		
		Day 7		Day 13			Day 7	Day 13	
		Oral	IV	Oral	IV	Oral	IV	Oral	IV
Firmicutes	Lachnospiraceae	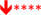	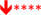	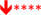	-	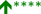	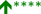	-	-
	Ruminococcaceae	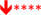	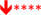	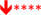	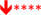	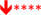	-	-	-
	Oscillospiraceae	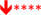	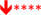	-	-	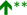	-	-	-
	Lactobacillaceae	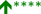	-	-	-	-	-	-	-
	Clostridiales_vadinBB60_group_fa	**‡_-_**	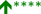	-	-	-	-	-	-
Bacteroidetes	Muribaculaceae	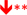	**‡_-_**	-	-	**‡_-_**	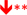	-	-
Proteobacteria	Enterobacteriaceae	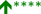	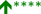	-	**‡_-_**	n.a.	n.a.	n.a.	n.a.
	Unclassified	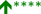	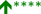	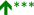	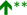	-	-	-	-

- = no significant difference, n.a. = taxon did not appear in sequencing results for this group,

‡ = data for this group were not normally distributed, therefore non-parametric tests were used.

## References

[1] Tang WHW, Kitai T, Hazen SL (2017). Gut Microbiota in Cardiovascular Health and Disease. Circ Res.

[2] Battson ML, Lee DM, Weir TL, Gentile CL (2018). The Gut Microbiota as a Novel Regulator of Cardiovascular Function and Disease. J Nutr Biochem.

[3] Leung C, Rivera L, Furness J, Angus P (2016). The Role of the Gut Microbiota in NAFLD. Nat Rev Gastroenterol Hepatol.

[4] Campo L, Eiseler S, Apfel T, Pyrsopoulos N (2019). Fatty Liver Disease and Gut Microbiota: A Comprehensive Update. J Clin Transl Hepatol.

[5] Lee CJ, Sears CL, Maruthur N (2019). Gut Microbiome and Its Role in Obesity and Insulin Resistance. Ann N Y Acad Sci.

[6] Lazar V, Ditu LM, Pircalabioru GG, Picu A, Petcu L, Cucu N, Chifiriuc MC (2019). Gut Microbiota, Host Organism, and Diet Trialogue in Diabetes and Obesity. Front Nutr.

[7] Sikalidis A, Maykish A (2020). The Gut Microbiome and Type 2 Diabetes Mellitus: Discussing a Complex Relationship. Biomedicines.

[8] Kowalski K, Mulak A (2019). Brain-Gut-Microbiota Axis in Alzheimer’s Disease. J Neurogastroenterol Motil.

[9] Westfall S, Lomis N, Kahouli I, SY D, Singh S, Prakash S (2017). Microbiome, Probiotics and Neurodegenerative Diseases: Deciphering the Gut Brain Axis. Cell Mol Life Sci.

[10] Rogers GB, Keating DJ, Young RL, Wong ML, Licinio J, Wesselingh S (2016). From Gut Dysbiosis to Altered Brain Function and Mental Illness: Mechanisms and Pathways. Mol Psychiatry.

[11] Cenit MC, Sanz Y, Codoñer-Franch P (2017). Influence of Gut Microbiota on Neuropsychiatric Disorders. World J Gastroenterol.

[12] Ducarmon QR, Zwittink RD, Hornung BVH, van Schaik W, Young VB, Kuijper EJ (2019). Gut Microbiota and Colonization Resistance against Bacterial Enteric Infection. Microbiol Mol Biol Rev.

[13] Pickard JM, Zeng MY, Caruso R, Núñez G (2017). Gut Microbiota: Role in Pathogen Colonization, Immune Responses, and Inflammatory Disease. Immunol Rev.

[14] Thursby E, Juge N (2017). Introduction to the Human Gut Microbiota. Biochem J.

[15] So D, Whelan K, Rossi M, Morrison M, Holtmann G, Kelly JT, Shanahan ER, Staudacher HM, Campbell KL (2018). Dietary Fiber Intervention on Gut Microbiota Composition in Healthy Adults: A Systematic Review and Meta-Analysis. Am J Clin Nutr.

[16] McBurney MI, Davis C, Fraser CM, Schneeman BO, Huttenhower C, Verbeke K, Walter J, Latulippe ME (2019). Establishing What Constitutes a Healthy Human Gut Microbiome: State of the Science, Regulatory Considerations, and Future Directions. J Nutr.

[17] Rinninella E, Raoul P, Cintoni M, Franceschi F, Miggiano GAD, Gasbarrini A, Mele MC (2019). What Is the Healthy Gut Microbiota Composition? A Changing Ecosystem across Age, Environment, Diet, and Diseases. Microorganisms.

[18] Fujio-Vejar S, Vasquez Y, Morales P, Magne F, Vera-Wolf P, Ugalde JA, Navarrete P, Gotteland M (2017). The Gut Microbiota of Healthy Chilean Subjects Reveals a High Abundance of the Phylum Verrucomicrobia. Front Microbiol.

[19] Lange K, Buerger M, Stallmach A, Bruns T (2016). Effects of Antibiotics on Gut Microbiota. Dig Dis.

[20] Kelly SA, Rodgers AM, Brien CO, Donnelly RF, Gilmore BF (2019). Gut Check Time: Antibiotic Delivery Strategies to Reduce Antimicrobial Resistance. Trends Biotechnol.

[21] Forslund K, Sunagawa S, Kultima JR, Mende DR, Arumugam M, Typas A, Bork P (2013). Country-Specific Antibiotic Use Practices Impact the Human Gut Resistome. Genome Res.

[22] Rolain JM (2013). Food and Human Gut as Reservoirs of Transferable Antibiotic Resistance Encoding Genes. Front Microbiol.

[23] Bengtsson-Palme J, Angelin M, Huss M, Kjellqvist S, Kristiansson E, Palmgren H, Larsson DGJ, Johansson A (2015). The Human Gut Microbiome as a Transporter of Antibiotic Resistance Genes between Continents. Antimicrob Agents Chemother.

[24] Kim S, Covington A, Pamer EG (2017). The Intestinal Microbiota: Antibiotics, Colonization Resistance, and Enteric Pathogens. Immunol Rev.

[25] Dethlefsen L, Relman DA (2011). Incomplete Recovery and Individualized Responses of the Human Distal Gut Microbiota to Repeated Antibiotic Perturbation. Proc Natl Acad Sci.

[26] Panda S, El Khader I, Casellas F, López Vivancos J, García Cors M, Santiago A, Cuenca S, Guarner F, Manichanh C (2014). Short-Term Effect of Antibiotics on Human Gut Microbiota. PLoS One.

[27] Palleja A, Mikkelsen KH, Forslund SK, Kashani A, Allin KH, Nielsen T, Hansen TH, Liang S, Feng Q, Zhang C, Pyl PT (2018). Recovery of Gut Microbiota of Healthy Adults Following Antibiotic Exposure. Nat Microbiol.

[28] Liu L, Wang Q, Wu X, Qi H, Das R, Lin H, Shi J, Wang S, Yang J, Xue Y, Mao D (2020). Vancomycin Exposure Caused Opportunistic Pathogens Bloom in Intestinal Microbiome by Simulator of the Human Intestinal Microbial Ecosystem (SHIME). Environ Pollut.

[29] Baümler AJ, Sperandio V (2016). Interactions between the Microbiota and Pathogenic Bacteria in the Gut. Nature.

[30] Leffler DA, Lamont JT (2015). Clostridium Difficile Infection. N Engl J Med.

[31] Jernberg C, Löfmark S, Edlund C, Jansson JK (2010). Long-Term Impacts of Antibiotic Exposure on the Human Intestinal Microbiota. Microbiology.

[32] Bartlett J (2002). Antibiotic-Associated Diarrhea. N Engl J Med.

[33] Zhang L, Huang Y, Zhou Y, Buckley T, Wang HH (2013). Antibiotic Administration Routes Significantly Influence the Levels of Antibiotic Resistance in Gut Microbiota. Antimicrob Agents Chemother.

[34] Birck M, Nguyen D, Cilieborg M, Kamal S, Nielsen D, Damborg P, Olsen J, Lauridsen C, Sangild P, Thymann T (2016). Enteral but Not Parenteral Antibiotics Enhance Gut Function and Prevent Necrotizing Enterocolitis in Formula-Fed Newborn Preterm Pigs. Am J Physiol Liver Physiol.

[35] Chantziaras I, Smet A, Haesebrouck F, Boyen F, Dewulf J (2017). Studying the Effect of Administration Route and Treatment Dose on the Selection of Enrofloxacin Resistance in Commensal Escherichia Coli in Broilers. J Antimicrob Chemother.

[36] Wiuff C, Lykkesfeldt J, Svendsen O, Aarestrup FM (2003). The Effects of Oral and Intramuscular Administration and Dose Escalation of Enrofloxacin on the Selection of Quinolone Resistance among Salmonella and Coliforms in Pigs. Res Vet Sci.

[37] Zeineldin M, Aldridge B, Blair B, Kancer K, Lowe J (2018). Impact of Parenteral Antimicrobial Administration on the Structure and Diversity of the Fecal Microbiota of Growing Pigs. Microb Pathog.

[38] De Smet J, Boyen F, Croubels S, Rasschaert G, Haesebrouck F, De Backer P, Devreese M (2018). Similar Gastro-Intestinal Exposure to Florfenicol After Oral or Intramuscular Administration in Pigs, Leading to Resistance Selection in Commensal Escherichia Coli. Front Pharmacol.

[39] Connelly S, Subramanian P, Hasan NA, Colwell RR, Kaleko M (2018). Distinct Consequences of Amoxicillin and Ertapenem Exposure in the Porcine Gut Microbiome. Anaerobe.

[40] Spor A, Koren O, Ley R (2011). Unravelling the Effects of the Environment and Host Genotype on the Gut Microbiome. Nat Rev Microbiol.

[41] Brinkman BM, Hildebrand F, Kubica M, Goosens D, Del Favero J, Declercq W, Raes J, Vandenabeele P (2011). Caspase Deficiency Alters the Murine Gut Microbiome. Cell Death Dis.

[42] Gu SL, Gong Y, Zhang J, Chen Y, Wu Z, Xu Q, Fang Y, Wang J, Tang LL (2020). Effect of the Short-Term Use of Fluoroquinolone and β-Lactam Antibiotics on Mouse Gut Microbiota. Infect Drug Resist.

[43] Ziegler M, Han JH, Landsburg D, Pegues D, Reesey E, Gilmar C, Gorman T, Bink K, Moore A, Kelly BJ (2019). Impact of Levofloxacin for the Prophylaxis of Bloodstream Infection on the Gut Microbiome in Patients with Hematologic Malignancy. Open Forum Infect Dis.

[44] Vacca M, Celano G, Calabrese FM, Portincasa P, Gobbetti M, De Angelis M (2020). The Controversial Role of Human Gut Lachnospiraceae. Microorganisms.

[45] Konikoff T, Gophna U (2016). Oscillospira: A Central, Enigmatic Component of the Human Gut Microbiota. Trends Microbiol.

[46] Ferrario C, Statello R, Carnevali L, Mancabelli L, Milani C, Mangifesta M, Duranti S, Lugli GA, Jimenez B, Lodge S, Viappiani A (2017). How to Feed the Mammalian Gut Microbiota: Bacterial and Metabolic Modulation by Dietary Fibers. Front Microbiol.

[47] Conway T, Cohen P (2015). Commenal and Pathogenic Ecoli Metabolism in the Gut. Microbiol Spectr.

[48] Kaur CP, Vadivelu J, Chandramathi S (2018). Impact of Klebsiella Pneumoniae in Lower Gastrointestinal Tract Diseases. J Dig Dis.

[49] Kurtz JR, Goggins JA, McLachlan JB (2017). Salmonella Infection: Interplay between the Bacteria and Host Immune System. Immunol Lett.

[50] Baker S, The HC (2018). Recent Insights into Shigella: A Major Contributor to the Global Diarrhoeal Disease Burden. Curr Opin Infect Dis.

[51] Zeng M, Inohara N, Nuñez G (2018). Mechanisms of Inflammation-Driven Bacterial Dysbiosis in the Gut. Mucosal Immunol.

[52] Garrett WS, Gallini CA, Yatsunenko T, Michaud M, Delaney ML, Punit S, Karlsson M, Bry L, Jonathan N, Gordon JI, Onderdonk AB (2011). Induce Spontaneous and Maternally Transmitted Colitis. Cell Host Microbe.

[53] Azad MAK, Sarker M, Li T, Yin J (2018). Probiotic Species in the Modulation of Gut Microbiota: An Overview. Biomed Res Int.

[54] Jones-Nelson O, Tovchigrechko A, Glover MS, Fernandes F, Rangaswamy U, Liu H, Tabor DE, Boyd J, Warrener P, Martinez J, Hilliard JJ (2020). Antibacterial Monoclonal Antibodies Do Not Disrupt the Intestinal Microbiome or Its Function. Antimicrob Agents Chemother.

[55] McAlister E, Dutton B, Vora LK, Zhao L, Ripolin A, Zahari DSZBPH, Quinn HL, Tekko IA, Courtenay AJ, Kelly SA, Rodgers AM (2020). Directly Compressed Tablets: A Novel Drug-Containing Reservoir Combined with Hydrogel-Forming Microneedle Arrays for Transdermal Drug Delivery. Adv Healthc Mater.

[56] Rodgers AM, McCrudden MT, Courtenay AJ, Kearney M-C, Edwards KL, Ingram RJ, Bengoechea J, Donnelly RF (2019). Control of *Klebsiella Pneumoniae* Infection in Mice Using Dissolving Microarray Patches Containing Gentamicin. Antimicrob Agents Chemother.

[57] Jakobsson HE, Jernberg C, Andersson AF, Sjölund-Karlsson M, Jansson JK, Engstrand L (2010). Short-Term Antibiotic Treatment Has Differing Long-Term Impacts on the Human Throat and Gut Microbiome. PLoS One.

[58] Caporaso JG, Lauber CL, Walters WA, Berg-Lyons D, Huntley J, Fierer N, Owens SM, Betley J, Fraser L, Bauer M, Gormley N (2012). Ultra-High-Throughput Microbial Community Analysis on the Illumina HiSeq and MiSeq Platforms. ISME J.

[59] Caporaso JG, Lauber CL, Walters WA, Berg-Lyons D, Lozupone CA, Turnbaugh PJ, Fierer N, Knight R (2011). Global Patterns of 16S RRNA Diversity at a Depth of Millions of Sequences per Sample. Proc Natl Acad Sci U S A.

[60] Schloss PD, Westcott SL, Ryabin T, Hall JR, Hartmann M, Hollister EB, Lesniewski RA, Oakley BB, Parks DH, Robinson CJ, Sahl JW (2009). Introducing Mothur: Open-Source, Platform-Independent, Community-Supported Software for Describing and Comparing Microbial Communities. Appl Environ Microbiol.

[61] Kozich JJ, Westcott SL, Baxter NT, Highlander SK, Schloss PD (2013). Development of a Dual-Index Sequencing Strategy and Curation Pipeline for Analyzing Amplicon Sequence Data on the Miseq Illumina Sequencing Platform. Appl Environ Microbiol.

[62] Pruesse E, Quast C, Knittel K, Fuchs BM, Ludwig W, Peplies J, Glöckner FO (2007). SILVA: A Comprehensive Online Resource for Quality Checked and Aligned Ribosomal RNA Sequence Data Compatible with ARB. Nucleic Acids Res.

[63] Quast C, Pruesse E, Yilmaz P, Gerken J, Schweer T, Yarza P, Peplies J, Glöckner FO (2013). The SILVA Ribosomal RNA Gene Database Project: Improved Data Processing and Web-Based Tools. Nucleic Acids Res.

[64] Edgar RC, Haas BJ, Clemente JC, Quince C, Knight R (2011). UCHIME Improves Sensitivity and Speed of Chimera Detection. Bioinformatics.

[65] R Core Team (2013). The R Project for Statistical Computing.

