# Supplementary material for: Antibiotic therapy and the gut microbiome: Investigating the effect of delivery route on gut pathogens
Source: ACS Infect Dis. Author manuscript; Available in PMC 2025 Sep 15. (PMC7618120; doi:10.1021/acsinfecdis.1c00081)
Supplement: Supplementary information [file EMS208027-supplement-Supplementary_information.docx]

Supporting Information

**Antibiotic therapy and the gut microbiome: Investigating the effect of delivery route on gut pathogens**

Stephen A. Kelly^1^,*,^‡^, Jonathan Nzakizwanayo^2^,^‡^, Aoife M. Rodgers^3^, Li Zhao^1^, Rebecca Weiser^4^ , Ismaiel A. Tekko^1,5^, Helen O. McCarthy^1^, Rebecca J. Ingram^6^, Brian V. Jones^2^, Ryan F. Donnelly^1^, Brendan F. Gilmore^1^

^1^ School of Pharmacy, Queen’s University Belfast, 97 Lisburn Road, UK, BT9 7BL

^2^ Department of Biology & Biochemistry, University of Bath, Claverton Down, Bath, UK, BA2 7AX

^3^ Department of Biology, Maynooth University, Maynooth, Co. Kildare, Ireland, W23 F2K8

^4^ Microbiomes, Microbes and Informatics Group, Organisms and Environment Division, Cardiff School of Biosciences, Cardiff University, UK, CF10 3AX

^5^ Department of Pharmaceutics and Pharmaceutical Technology, Faculty of Pharmacy, Aleppo University, Aleppo, Syria

^6^ Wellcome-Wolfson Institute for Experimental Medicine, School of Medicine, Dentistry and Biomedical Sciences, Queen’s University Belfast, 97 Lisburn Road, UK, BT9 7BL

^*^ Corresponding author. Email: stephen.kelly@qub.ac.uk

^‡^ These authors contributed equally to the work.

A

B

**Figure S1**. Diversity analysis before (Day 5a) and after (Day 7) antibiotic administration measured by InvSimpson Index, showing untreated, oral and IV groups. A. Amoxicillin. B. Levofloxacin. **** = p<0.0001, *** = p<0.0002, ** = p<0.0021, * = p<0.0332.

1. *Mothur command scripts used to run amoxicillin samples*

make.contigs(ffastq=JN_Amox_NOV2020_16S_S1_L001_R1_001.fastq, rfastq=JN_Amox_NOV2020_16S_S1_L001_R2_001.fastq, rindex=JN_Amox_NOV2020_16S_S1_L001_I1_001.fastq, oligos=Jon_Amox_noDay9_IV4_14Nov20.oligos, checkorient=t, bdiffs=1, trimoverlap=T, processors=8)

summary.seqs(fasta=current, processors=8)

screen.seqs(fasta=current, group=current, contigsreport=current, minlength=252, maxlength=253, maxhomop=7, maxambig=0, mismatches=1, maxn=0, processors=8)

summary.seqs(fasta=current)

unique.seqs(fasta=current)

count.seqs(name=current, group=current)

summary.seqs(fasta=current, count=current, processors=8)

align.seqs(fasta=current, reference=silva138.v4.align)

summary.seqs(fasta=current, count=current)

screen.seqs(fasta=current, count=current, summary=current, start=1968, end=11550, maxhomop=7)

filter.seqs(fasta=current, vertical=T, trump=.)

unique.seqs(fasta=current, count=current)

pre.cluster(fasta=current, count=current, diffs=2)

chimera.uchime(fasta=current, count=current, dereplicate=t)

remove.seqs(fasta=current, accnos=current, name=current)

summary.seqs(fasta=current, count=current)

classify.seqs(fasta=current, count=current, reference=silva138.v4.align, taxonomy=silva.nr_v138.tax, cutoff=80)

remove.lineage(fasta=current, count=current, taxonomy=current, taxon=Chloroplast-Mitochondria-unknown-Archaea-Eukaryota)

summary.tax(taxonomy=current, count=current)

remove.groups(fasta=JN_Amox_NOV2020_16S_S1_L001_R1_001.trim.contigs.good.unique.good.filter.unique.precluster.pick.pick.fasta, taxonomy=JN_Amox_NOV2020_16S_S1_L001_R1_001.trim.contigs.good.unique.good.filter.unique.precluster.pick.nr_v138.wang.pick.taxonomy, count=JN_Amox_NOV2020_16S_S1_L001_R1_001.trim.contigs.good.unique.good.filter.unique.precluster.denovo.uchime.pick.pick.count_table, groups=115_Day6_MN1-116_Day6_MN2-117_Day6_MN3-118_Day6_MN4-119_Day6_MN5-120_Day6_MN6-139_Day7_MN1-140_Day7_MN2-141_Day7_MN3-142_Day7_MN4-143_Day7_MN5-144_Day7_MN6-163_Day8_MN1-164_Day8_MN2-165_Day8_MN3-166_Day8_MN4-167_Day8_MN5-168_Day8_MN6-187_Day9_MN1-188_Day9_MN2-189_Day9_MN3-190_Day9_MN4-191_Day9_MN5-192_Day9_MN6-19_Day1_MN1-20_Day1_MN2-211_Day12_MN1-212_Day12_MN2-213_Day12_MN3-214_Day12_MN4-215_Day12_MN5-216_Day12_MN6-21_Day1_MN3-22_Day1_MN4-235_Day13_MN1-236_Day13_MN2-237_Day13_MN3-238_Day13_MN4-239_Day13_MN5-23_Day1_MN5-240_Day13_MN6-24_Day1_MN6-43_Day2_MN1-44_Day2_MN2-45_Day2_MN3-46_Day2_MN4-47_Day2_MN5-48_Day2_MN6-67_Day5a_MN1-68_Day5a_MN2-69_Day5a_MN3-70_Day5a_MN4-71_Day5a_MN5-72_Day5a_MN6-91_Day5b_MN1-92_Day5b_MN2-93_Day5b_MN3-94_Day5b_MN4-95_Day5b_MN5-96_Day5b_MN6-0_MOCK-243_MOCK-244_MOCK-241_KITOME-129_Day7_Or3)

summary.tax(taxonomy=current, count=current)

cluster.split(fasta=current, count=current, taxonomy=current, splitmethod=classify, taxlevel=4, cutoff=0.03, processors=8)

make.shared(list=current, count=current, label=0.03)

classify.otu(list=current, count=current, taxonomy=current, label=0.03)

rename.file(fasta=current, column=current, shared=current, list=current, count= current, taxonomy=current, constaxonomy=current, prefix=JN_Amox_Nov2020_final)

count.groups(shared=current)

sub.sample(shared=current, size=2562)

rarefaction.single(shared=JN_Amox_Nov2020_final.opti_mcc.shared, calc=sobs, freq=100)

summary.single(shared=JN_Amox_Nov2020_final.opti_mcc.shared, calc=nseqs-sobs-chao-ace-invsimpson-npshannon-shannon-coverage, subsample=2562)

1. ***Mothur command scripts used to run levofloxacin samples***

make.contigs(ffastq=JN_Levo_OCT2020_16S_S1_L001_R1_001.fastq, rfastq=JN_Levo_OCT2020_16S_S1_L001_R2_001.fastq, rindex=JN_Levo_OCT2020_16S_S1_L001_I1_001.fastq, oligos=Jon_Levo_noDay9_IV4_15Nov20.oligos, checkorient=t, bdiffs=1, trimoverlap=T, processors=8)

summary.seqs(fasta=current, processors=8)

screen.seqs(fasta=current, group=current, contigsreport=current, minlength=252, maxlength=253, maxhomop=7, maxambig=0, mismatches=1, maxn=0, processors=8)

summary.seqs(fasta=current)

unique.seqs(fasta=current)

count.seqs(name=current, group=current)

summary.seqs(fasta=current, count=current, processors=8)

align.seqs(fasta=current, reference=silva138.v4.align)

summary.seqs(fasta=current, count=current)

screen.seqs(fasta=current, count=current, summary=current, start=1968, end=11550, maxhomop=7)

filter.seqs(fasta=current, vertical=T, trump=.)

unique.seqs(fasta=current, count=current)

pre.cluster(fasta=current, count=current, diffs=2)

chimera.uchime(fasta=current, count=current, dereplicate=t)

remove.seqs(fasta=current, accnos=current, name=current)

summary.seqs(fasta=current, count=current)

classify.seqs(fasta=current, count=current, reference=silva138.v4.align, taxonomy=silva.nr_v138.tax, cutoff=80)

remove.lineage(fasta=current, count=current, taxonomy=current, taxon=Chloroplast-Mitochondria-unknown-Archaea-Eukaryota)

summary.tax(taxonomy=current, count=current)

remove.groups(fasta=JN_Levo_OCT2020_16S_S1_L001_R1_001.trim.contigs.good.unique.good.filter.unique.precluster.pick.pick.fasta, taxonomy=JN_Levo_OCT2020_16S_S1_L001_R1_001.trim.contigs.good.unique.good.filter.unique.precluster.pick.nr_v138.wang.pick.taxonomy, count=JN_Levo_OCT2020_16S_S1_L001_R1_001.trim.contigs.good.unique.good.filter.unique.precluster.denovo.uchime.pick.pick.count_table, groups=115_Day6_MN1-116_Day6_MN2-117_Day6_MN3-118_Day6_MN4-119_Day6_MN5-120_Day6_MN6-139_Day7_MN1-140_Day7_MN2-141_Day7_MN3-142_Day7_MN4-143_Day7_MN5-144_Day7_MN6-163_Day8_MN1-164_Day8_MN2-165_Day8_MN3-166_Day8_MN4-167_Day8_MN5-168_Day8_MN6-187_Day9_MN1-188_Day9_MN2-189_Day9_MN3-190_Day9_MN4-191_Day9_MN5-192_Day9_MN6-19_Day1_MN1-20_Day1_MN2-211_Day12_MN1-212_Day12_MN2-213_Day12_MN3-214_Day12_MN4-215_Day12_MN5-216_Day12_MN6-21_Day1_MN3-22_Day1_MN4-235_Day13_MN1-236_Day13_MN2-237_Day13_MN3-238_Day13_MN4-239_Day13_MN5-23_Day1_MN5-240_Day13_MN6-24_Day1_MN6-43_Day2_MN1-44_Day2_MN2-45_Day2_MN3-46_Day2_MN4-47_Day2_MN5-48_Day2_MN6-67_Day5a_MN1-68_Day5a_MN2-69_Day5a_MN3-70_Day5a_MN4-71_Day5a_MN5-72_Day5a_MN6-91_Day5b_MN1-92_Day5b_MN2-93_Day5b_MN3-94_Day5b_MN4-95_Day5b_MN5-96_Day5b_MN6-0_MOCK-243_MOCK-244_MOCK-241_KITOME-245_KITOME-246_KITOME-129_Day7_Or3)

summary.tax(taxonomy=current, count=current)

cluster.split(fasta=current, count=current, taxonomy=current, splitmethod=classify, taxlevel=4, cutoff=0.03, processors=8)

make.shared(list=current, count=current, label=0.03)

classify.otu(list=current, count=current, taxonomy=current, label=0.03)

rename.file(fasta=current, column=current, shared=current, list=current, count= current, taxonomy=current, constaxonomy=current, prefix=JN_Levo_Dec2020_final)

count.groups(shared=current)

sub.sample(shared=current, size=3994)

rarefaction.single(shared=JN_Levo_Dec2020_final.opti_mcc.shared, calc=sobs, freq=100)

summary.single(shared=JN_Levo_Dec2020_final.opti_mcc.shared, calc=nseqs-sobs-chao-ace-invsimpson-npshannon-shannon-coverage, subsample=2562)

***c) Example R Scripts: NMDS ordination of Bray-Curtis dissimilarity distances for samples in different treatment groups. Scripts for amoxicillin Day 5a vs Day 7 shown.***

**#Load packages**

library(vegan)

library(RVAideMemoire)

**#Read in data**

#OTU table (OTUs in column 1, Samples with read numbers in other columns)

#Load Amox_OTU_5_7.txt

data<-read.table(file.choose(), header=T)

#Metadata (Samples in column 1, metadata in other columns)

#Load Amox_OTU_5_7_env.txt

meta_table<-read.csv(file.choose(),row.names=1,check.names=FALSE)

**#Transform OTU table**

abund_table<-t(data)

**#Check levels of metadata column ‘Code’ (the groupings to test), re-order and make into a factor**

meta_table$Code

#IV_5 IV_7 O_5 O_7 Un_5 Un_7

meta_table$Code <- factor(meta_table$Code, levels =c("Un_5","Un_7","O_5","O_7","IV_5","IV_7"))

meta_table$Code

env.code<-as.factor(meta_table$Code)

**#Bray-Curtis analysis**

BC_OTU_table <- vegdist(abund_table, method="bray", binary = FALSE)

mat_BC <- data.matrix(BC_OTU_table)

**#NMDS**

sol<-metaMDS(abund_table,distance = "bray", k = 2, trymax = 50)

sol

**#Look at stressplot**

plot(sol, type="points")

stressplot(sol)

**#Choose palette for plots**

mypalette<-c("#969696","#252525","#74c476","#006d2c","#9e9ac8","#54278f")

palette(mypalette)

**#Draw plot, add points and groupings**

win.graph(18,18)

plot(sol, display="sites", type="n")

points(sol, cex=1.2, pch=19, col=env.code)

ordiellipse(sol, group=env.code, show.groups = "Un_5", col="#969696", kind="sd", lwd=2)

ordiellipse(sol, group=env.code, show.groups = "Un_7", col="#252525", kind="sd", lwd=2)

ordiellipse(sol, group=env.code, show.groups = "O_5", col="#74c476", kind="sd", lwd=2)

ordiellipse(sol, group=env.code, show.groups = "O_7", col="#006d2c", kind="sd", lwd=2)

ordiellipse(sol, group=env.code, show.groups = "IV_5", col="#9e9ac8", kind="sd", lwd=2)

ordiellipse(sol, group=env.code, show.groups = "IV_7", col="#54278f", kind="sd", lwd=2)

legend("topright", c("Un_5a","Un_7","Or_5a","Or_7","IV_5a","IV_7"), cex=0.9, col=c ("#969696","#252525","#74c476","#006d2c","#9e9ac8","#54278f"), lwd=2)

**#Statistical analysis**

#Permanova to determine significant difference between all groups

adonis(BC_OTU_table ~ Code, data = meta_table, permutations = 999)

#Test beta dispersion as a condition of permanova, the result should be non-significant to demonstrate homogeneity of variances

beta <-betadisper(BC_OTU_table, meta_table$Code)

permutest(beta)

#Post-hoc pairwise tests to determine significant differences between groups

pairwise.perm.manova(BC_OTU_table , meta_table$Code, nperm = 999, p.method = "BY")

**Supplementary Table 1. Statistical analysis of treatment groups for untreated and amoxicillin sample groups at Day 5a and Day 7.**


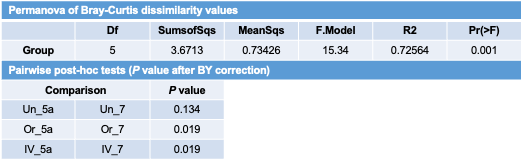


Footnotes: Untreated group Day 5a (Un_5a) and Day 7 (Un_7); Oral administration group Day 5a (Or_5a) and Day 7 (Or_7); IV administration group Day 5a (IV_5a) and Day 7 (IV_7)

**Supplementary Table 2. Statistical analysis of treatment groups for untreated and levofloxacin sample groups at Day 5a and Day 7.**


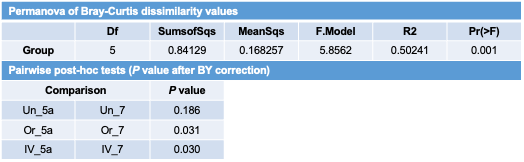


Footnotes: Untreated group Day 5a (Un_5a) and Day 7 (Un_7); Oral administration group Day 5a (Or_5a) and Day 7 (Or_7); IV administration group Day 5a (IV_5a) and Day 7 (IV_7)


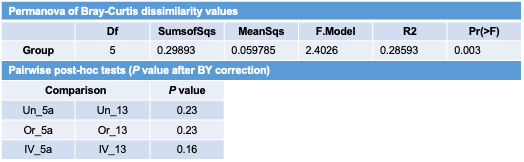

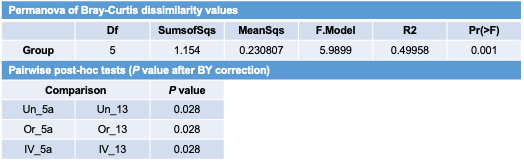


**Supplementary Table 3. Statistical analysis of treatment groups for untreated and amoxicillin sample groups at Day 5a and Day 13.**

**Supplementary Table 4. Statistical analysis of treatment groups for untreated and levofloxacin sample groups at Day 5a and Day 13.**

Footnotes: Untreated group Day 5a (Un_5a) and Day 13 (Un_13); Oral administration group Day 5a (Or_5a) and Day 13 (Or_13); IV administration group Day 5a (IV_5a) and Day 13 (IV_13)

Footnotes: Untreated group Day 5a (Un_5a) and Day 13 (Un_13); Oral administration group Day 5a (Or_5a) and Day 13 (Or_13); IV administration group Day 5a (IV_5a) and Day 13 (IV_13)
